# The impact of participatory action research and endogenous integrated soil fertility management on farm-gate dietary outputs in northern Tanzania

**DOI:** 10.1016/j.heliyon.2023.e21888

**Published:** 2023-11-05

**Authors:** Powell Mponela, Julius Manda, Michael Kinyua, Job Kihara

**Affiliations:** a Alliance for Bioversity and CIAT, Duduville Campus, Nairobi, Kenya; b International Institute of Tropical Agriculture (IITA), C/O World Vegetable Centre, Arusha, Tanzania

**Keywords:** *Participatory action research*, *Soil fertility management*, *Extended regression model*, *Impact*, *Food and nutrition security*

## Abstract

In most developing countries, although agricultural extension and research devolved since 1980s to promote relevance, cost-effectiveness, ownership, and sustainability, participatory action research (PAR) have been run, albeit with limited empirical evidence on their impacts on farmers livelihoods. The study use a three-stage extended regression model (ERM) to estimate the effect PAR and the promoted agronomic practices on crop produce dietary outputs considering potential endogenous engagement, omitted variable bias, reverse causality, endogenous covariates, and factor simultaneity. Using a sample of 607 small family farms with varying levels of research engagement in the Northern Highlands of Tanzania, the study found that PAR was associated with increased farm-gate dietary outcomes: per-capita calories, proteins, and minerals produced by 139, 216, and 143 %, respectively, and consumption of farm-produced minerals by 74–200 %. The organic manure application was associated with increased the dietary outputs by 62–113 % while the application of inorganic fertilizer with increased protein and mineral outputs by 68 and 105 %, respectively. The crop diversification was associated with increased dietary outputs by 11–25 % while pest and diseases control with increased dietary outputs by 12–17 % but with reduced consumption by 13–14 %. Soil and water conservation measures including terracing were associated with decreased dietary outputs. These findings indicate that PAR contributes to nutritional outcomes of smallholder farmers contingent on the promotion of low-cost input sources and crop diversification which can be leveraged to inform upscaling of participatory policies, strategies, and technologies.

## 1 Introduction

In sub-Sahara Africa, the dietary outcomes under smallholder farming systems are affected by climate change, the continued reliance on rainfed farming, a growing population, and low mitigation and adaptive capacities. To improve adaptive capacities, agricultural extension has been pivotal in enhancing the adoption of SI and good agronomic practices but with reduction in government extension services, strategies to educate farmers through their participation in action research and demonstrations in farmers settings have become quintessential research and extension approach. There is growing evidence that participatory action research (PAR) unlocks adoption of improved agricultural technologies, crop productivity and net returns [[1], [2], [3], [4], [5]]. While there are several studies on the adoption and productivity effects of participation, fewer studies have explored the linkage between participation in PAR, the adoption of technologies and food security outcomes [6]. Over the past 3 decades, a participatory action research approach, ‘*mother-baby*’, has been widely used to promote integrated soil fertility management components including improved varieties, inorganic fertilizer, organic manure, soil erosion control and water conservation measures on smallholder farms in Africa and Asia [2,7]. Hence impact pathways for PAR is wide: ranging from guiding variety selection [8,9], evaluating technical performance of the combined soil fertility management (SFM) strategies [10,11], to resolving institutional conflicts over tenure and social safety nets [11]. In addition to technological enhancements, farmers gain technical expertise in broader agronomic practices through researcher-farmer co-designed, farmer-managed learning ‘*mother*’ trials that are established in a centralized, easily accessible location where the performance of SFM technologies and agronomic practices are tested against their local practices and demonstrated to for fitting in microclimates and household farm conditions in the wider community in “*baby*” trials [11,12].

Sustaining productivity is the primary goal for smallholders of maize-mixed farming system which is most prevalent in eastern and southern Africa [13] as climate change induced productivity decline of up to 37 % is projected by 2050 [[14], [15], [16]]. In spite of the risks, the region's population increasingly rely on maize for up to 40 % of the 2045 daily caloric consumption [17,18]. Over the past 6 decades, innovations have been availed and efforts are ongoing to increase adoption [[19], [20], [21], [22]]. Studies show that engagement in participatory action research enhances adoption of technologies [5], is a pathway towards climate change adaptation [11] and induces diversity in diets [22] and most important for achievement of children's minimum dietary requirements [23]. In addition to availing technologies, participatory action research provides nutritional education which has also been found to improve nutrition outcomes [24] though evidence in the past decade has been scanty.

This study aimed to examine the impact of participation in action research and adoption of improved agronomic technologies on the production and consumption of calories, proteins, and minerals (hereinafter “dietary outcomes”). Notwithstanding that several studies have examined the impact of agricultural interventions on dietary diversity, relatively few studies have specifically considered the impact on dietary outcomes. In most developing countries, the inadequacies of these minerals are responsible for many health problems such as malnutrition and low immunity, especially in children [25]. As farmers seek to improve their faming conditions in a community, they explore a combination of agro-advisory sources including classical extension services, participatory approaches, media and more recently, digital mobile solutions. The engagement in action research could either be a complimentary or an alternative pedagogical approach for adult learning through action [26]. Bearing this in mind, it is assumed that those not participating in action research rely on other knowledge sources [26]. Second, a three-stage simultaneous equation model is used to disentangle the relationship among PAR, the adoption of SFM and nutrition. Specifically, the extended regression model (ERM) is used to control for both observed and unobserved heterogeneity to consistently estimate the impact. The first stage models the PAR in an ordered choice regression framework. The second stage accounts for the endogeneity of the decision to adopt SFM technologies, whereas the last stage uses the ordinary least squares regression (OLS) to estimate the impact of participation and adoption on dietary outcomes.

The rest of the article is organized as follows: The next section outlines the methodology which includes the sampling and empirical strategies. Section 3 presents the descriptive and empirical results, while the penultimate section provides the discussion. The last section presents the conclusion and policy recommendations.

## 2 Materials and methods

### 2.1 Study site and sampling

**Fig. 1:**
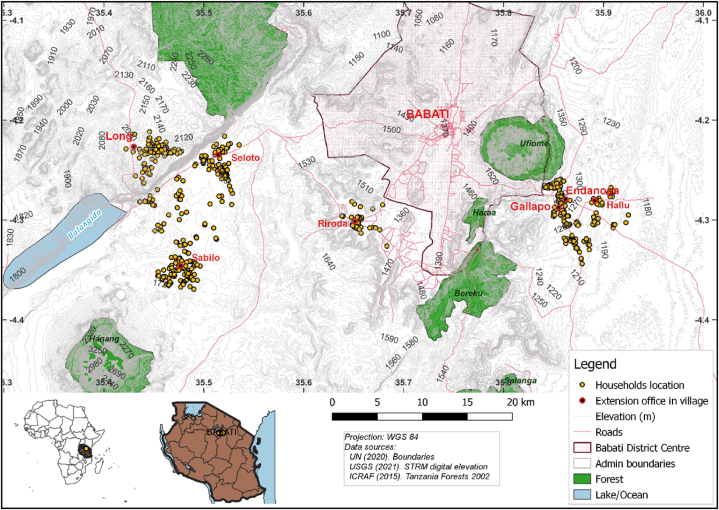
Distribution of sampled farming households in Babati District, Northern Tanzania.

In Tanzania, approximately 4.96 million smallholder farmers cultivate 80 % of farms averaging 1.2 ha and produce 70 % of the food consumed [27]. The study was conducted in Babati District situated in the Northern Highlands (Fig. 1) with undulating terrain (elevation from 1180 to 2170 m) and annual rainfall regime ranging from unimodal (750–900 mm) to bimodal (1200 mm) across diverse agro-ecological zones that provide a productive potential for diversified livelihood strategies [28]. The population pressure is eminent with an annual growth rate of 3.8 % and more than 70 % rural-based that rely on farming as the primary livelihood. Since the area has an archetypical historic record of agro-advisory services, agricultural potential, and technology deployment, it has been an action research use case for the past 8 years [2]. Maize-pigeon pea intercrop is the most common farming system in the area where maize is mostly grown for home consumption while pigeon peas are consumed while green but mostly sold to export markets when dry [29,30]. Farmers mostly press sunflower oil for home consumption and sell the seed cakes [31]. Few farmers intercrop common beans with maize for home consumption but the majority plant beans as the sole crop earmarked for the local and regional markets [32]. To improve diets, the Africa RISING project promoted crop diversification by integrating traditional vegetables that offer higher nutritional benefits into the maize-legume systems [33]. Consumption of animal products is low in Babati, hence plant-based diets are promoted to provide nutritional and health benefits [34]. To study outputs from mixed cropping systems, some studies aggregate produce value [35,36], food expenditure [37], crop income [38] but in this study, a dietary outputs is used which has implications on household food and nutrition security.

The sample of smallholder farming households was drawn using multi-stage purposive and stratified random sampling. First, the villages: Long and Seloto where biophysical characterisation was done in 2013 [29]; Gallapo, Riroda, and Sabilo where action research was implemented, plus Endanoga and Hallu that were reached during the out-scaling demonstration were purposely chosen. To evaluate impact of participation in PAR, the sampling frame included three strata: the 2.4 % of the community who participated actively in action research on “*mother*” trials and received improved seeds and fertilizer for trials plus intensive training by researchers and extension agents, the 10.1 % who participated in demonstration as “*babies*” and received improved inputs for demonstration plus brief training by extension agents, and the 87.7 % who did not actively participate but attended community sensitisation meetings and field days, passively observed the trails that were set along community roads, and could have accessed information through local networks. Considering that the study aims to evaluate multiple technologies with multiple covariates and outcomes, the sample size, *n*, was adjusted for design effects [39] and estimated as (Eq. (1)).

(1) $$n=\left[z^{2}*\frac{p*q}{e^{2}}\right]*d*r$$

where *z* = standard normal distribution linked to 95 % confidence interval, *p* = expected prevalence was set at 50 % which yields the largest sample size to capture wide adoption (86 % manure that include animal droppings, household/farm residues and compost that are transported to the field) as well as low adoption (11 % inorganic fertilizers) [30]; *q* = 1-*p* (expected non-prevalence); e = relative desired precision was set at 5 % to capture low usage; *d* = design effect was set at 1.3. To account for heterogeneity between the strata due to engagement levels; and *r* = non-response rate of 5 %. A stratified random sample of 607 family farms was randomly drawn disproportionate to strata size (∼200 from each stratum) but proportionate to village populations which represented 4.9 % of the household farm population in the region and 9.9 % of target villages.

### 2.2 Empirical strategy

Due to non-random enrolment in the action research program and potential change in farmer behaviours and contexts, the study used a cross-sectional survey and subject matter knowledge to establish the causal and temporal ordering of variables [40,41]. The participatory action research hinges on the development of human capital, enhances capacity to question the knowledge, opens opportunities for novelty, and affirms that experience can be a basis for knowing and that experimental learning can lead to a legitimate form of knowledge that influences practice [26,42]. In this study, action research was implemented to capacitate smallholder farmers in designing, implementing, and evaluating on-farm soil fertility management research to enhance and sustain their farm productivity. The project assumed that farmers participate in action research on the “*mother*” trial and adopt SFM components as “*babies”* to improve their welfare in terms of, among others, food, and nutrition security.

In theory, considering PAR as an exogenous program and given that I* is the difference between utility if farmer, *i*, participates (*U*_Pi1_) and the utility from non-participation (*U*_*Pi0*_), the decision to participate *P* is made when *I*_*i*_ = *U*_*Pi1*_ – *U*_*Pi0*_ > 0. Since the utility is unobservable, it can be expressed as the function of observable attributes in the latent variable estimated using the ordered probit model [43] as (Eq. (2)):

(2) $$P_{i}^{*}={\beta x1}_{i}+\varepsilon_{i1}$$

where *β* is a vector of parameters to be estimated; *x1* is a vector of household and farm attributes that are key in framing farmers' capacity to participate in a program including education level among household members as a proxy for technical understanding, age of head as a proxy for decision making, labour and farm size as resource endowments, and income from non-crop activities indicating competing/complementing livelihood strategies [37,44]; and ε is the random error term.

Secondly, given a set of soil improvement options that include inorganic fertilizers and organic manures (denoted *F*) that were promoted under the action research, the probability for a farmer to adopt the *F* was assumed to be related to the expected utility derived from its realised and future potential outcomes. Given that I* is the difference between utility if farmer, *i*, adopts SFM (U_Fi1_) and the utility from non-adoption (*U*_*Fi0*_), such that the decision to adopt *F* is made when *I*_*Fi*_ = *U*_*Fi1*_ – *U*_*Fi0*_ > 0. In this case, the unobserved utility can be expressed as the function of observable attributes in the latent variable estimated using the probit model [43] and expressed as (Eq. (3)):

(3) $$F_{i}^{*}=P_{i}+{\beta x2}_{i}+\varepsilon_{i2}\;\text{with}\;F_{i}=\left\{ \begin{array}{c} 1\;\text{if}F_{i}^{*}>0\\ 0\;\text{otherwise} \end{array}\right.$$

Where *F* is the binary: 1 = adopt and 0 if otherwise; *x2* are household and farm features that constrain or enable adoption [45,46]. *P* influences crop productivity through *F* and directly through the good agricultural practices learned and can be expressed as linear determinants of the farm-gate nutritional outcomes, *Y*_*s*_, in an ordinary least squares (OLS) model as (Eq. (4)):

(4) $$Y_{si}=\alpha {x3}_{i}+\delta F_{ij}+P_{i}+\varepsilon_{i3}$$

The farm-gate nutritional outcomes include gross energy and crude protein and macro-minerals which were calculated using the food composition for the 13 crops and fruits [47]. The minerals included calcium, magnesium, phosphorus, potassium, sodium, sulphur, and chloride. To account for significant differences in household sizes, per capita production and consumption were calculated. *X3* are other yield and nutritional controlling agronomic practices including crop composition, soil and water conservation, weeding, pest and disease control, labour input, and farm size.

This model fit requires that there be no confounding variables correlated with *x*_*s*_, *F* and *P*; *x*_*s*_, *F* and *P* be measured without error, there be no reverse causation (*x*_*s*_, *P* and *F*_*s*_ affect Y, but Y must not affect *x*_*s*_, *P* and *F*_*s*_), and *x*_*s*_, *P* and *F*_*s*_ not correlated with *ε* [43]. However, in bottom-up participatory approaches, technology acquisition and adoption are non-random. Furthermore due to extension regime changes and the technology boom and bust of the 1980s–90s and post 2000 participatory soil management projects including land management program (LAMP) [48], *P* and *F*_s_ are potentially primed by previous nutritional outcomes, i.e. having an inherent reverse causality. Moreover, there are considerable measurement errors in the farmer reports during surveys that would lead to attenuation bias. Furthermore, there exist heterogeneous treatment effects as *F*_*s*_ are adapted to land parcels with different fertility and response, compounded by differences in management practices. Therefore, the error terms of equations (1), (2), (3)) may be correlated which may lead to biased estimates.

These are key challenges to ascertaining the validity of causal inference made with observational program impact evaluation [41]. Bias arising from observables are controlled by including farmer, plot, and institutional variables. Unmeasured confounders such as ability and motivation that participation in action research fosters are difficult to control but may influence the decision of the households to participate/adopt SFM and become part of the error term thereby leading to biased estimates. Propensity score matching (PSM) and instrumental variables (IV) are the main approaches used in program evaluation [43]. The PSM approach is used when it is practical to collect data on every confounding variable to ensure a balance of observed variables between treatment and control groups. However, the PSM approach only controls for observed and not unobserved heterogeneity. In our study, the decision to participate and to adopt SFM was not random as such farmers may have self-selected into the adoption category based on their potential gains thereby leading to endogeneity problems. To properly account for self-selection, reverse causality and omitted variable bias, the quasi-experimental method is used - instrumental variables (IV) – which is most suited [43,[49], [50], [51]].

To control for the endogeneity of participation and SFM adoption, *agent-distance* is used as an IV. The variable measures the distance between the global positioning system (GPS) location of households to the six extension agent offices spread across the study area. It is assumed that it gives the farmers in proximity an engagement advantage but does not influence nutritional outcomes directly [52]. The instrument further addresses the omitted variable bias problem as *agent-distance* is assumed to be uncorrelated with the farmer's resource endowments and farm productivity and captures cross-sectional random variations [53]. Several studies have used distance as a valid IV [[54], [55], [56]], but unlike these studies, GPS was used to accurately measure the distance which in turn increases the credibility of the IV [57].

The GPS is further used to estimated elevation that captures variations across ecological zones from medium-altitude low-rainfall to high-altitude high-rainfall to instrument the decision to adopt SFM technologies [57]. Mindful of potential information dropout as farmers skip seasons for some SFM components such as manure with residual effects, non-usage could not be construed as non-adoption. Hence, frequency of manure usage in the previous five years was captured. Decisions to adopt or not could arise from substitutional and complementary effects of the SFM components as farmers continuously explore options. SFM components, therefore, have a simultaneous causality.

To address the potential endogenous participation in action research, endogenous covariates, and capture simultaneity, the extended regression model is used [58]. The ERM is estimated in 3 stages [59]: the first and second stages focus on exogenous variables to estimate probabilities for participation and adoption (Eq. (5) and Eq. (6)), while the third stage uses the endogenous variables as predictors of the *m*th farm level dietary indicator (Eq. (7)).

(5) $$\text{Participation}\;\text{in}\;\text{action}\;\text{research}P_{i}^{*}=\beta_{0}+\beta_{1}{x1}_{i}+{\beta_{2}z_{i}+\beta }_{2}{IV}_{i}{+\beta }_{3}\pi_{i}+\varepsilon_{i}$$

(6) $$\text{Endogenous}\left(\text{SFM}\right)\;F_{ij}^{*}=\beta_{0j}+\beta_{1}{x2}_{ij}+\beta_{2}z_{i}+{\beta_{2}IV}_{i}+\beta_{3}\pi_{ij}+\varepsilon_{ij}$$

(7) $$\text{Outcome}\left(\text{dietary}\;\text{value}\right)\;{\log(Y}_{im})=\beta_{0m}+\beta_{1}{x3}_{im}+\beta_{2}z_{i}+\beta_{2}P_{i}+{\beta_{3}F}_{i}+\varepsilon_{im}$$

where *IV* is the *agent-distance* instrumental variable, π is the landscape elevation, which is used to account for random factors across spatial scales. To address reverse causality, IVs, *z*, (livestock resources and media information sources) are used in treatment, endogenous and outcome variables. It is assumed that the livestock resources affect organic manure availability and adoption and hence only indirectly influence crop productivity. The prevailing media information access is assumed to influence knowledge of fertilizers which is being advertised on radios as well as relay information on action research but does not directly influence crop productivity.

Given that treatment, *P* is ordinal, the average treatment effect on the treated (ATT) that measure the impact of participation in action research is estimated as (Eq. (8)):

(8) $$ATT=E\left(y_{pi}|P,F,x,z\right)-E\left(y_{0i}|P,F,x,z\right)$$

where *y*_*p*_ is the expected dietary value realised by farmers belonging to *p*th [1,2] participation level [1 = demonstration as “*baby”* trial, 2 = action research on “*mother*” trial] and *y*_*0*_ is the expected dietary value if the farmer had chosen not to participate, which is estimated as a counterfactual reference.

## 3 Results

### 3.1 Descriptive summary

Farming in the area is both commercial and subsistence with a considerable proportion of own production consumed (37 % of maize, 46 % sunflower, 18 % beans, and 42 % pigeon peas) while the greater part is sold (Table 1). Farmers mostly combine 2–4 crops including maize (86 %), pigeon peas (68 %), beans (54 %), sunflower (28 %), cowpea (22 %), fruits (22 %), potatoes (15 %), garden peas (12 %), and bananas, lablab, sweet potatoes, sorghum, pumpkins, cassava, groundnuts, chickpea, and tomatoes grown by less than 10 %. In addition to maize, farmers obtain dietary inputs from consumption of own produced groundnuts, pigeon peas, and lablab.

**Table 1:** Annual average energy (MJ), protein (kg) and minerals (g) produced and consumed per household.

Crop	production				consumption			
	energy	protein	minerals	n	energy	protein	minerals	n
Maize	48,011	204	22,218	540	17,776	76	8226	535
Sorghum	15,798	91	8319	24	4835	28	2546	7
Sunflower	10,210	57	8029	174	4677	26	3678	156
Groundnuts	20,828	210	10,449	3	7863	79	3945	2
Beans	10,685	142	15,224	324	1935	26	2757	281
Pigeon peas	9970	119	9810	418	4191	50	4123	255
Chickpea	18,064	206	17,511	139	835	9	809	7
Cowpea	808	11	976	27	291	4	352	23
Lablab	2660	27	6430	28	1951	20	4716	2
Irish potatoes	6427	41	10,979	89	1399	9	2389	43
Sweet potatoes	5951	16	4651	3	744	2	581	2
Cassava	5525	8	3748	8	691	1	469	8
Banana	4989	15	6418	134	1439	4	1851	133
Fruits	1877	7	861	33	385	1	176	32

**Fig. 2:**
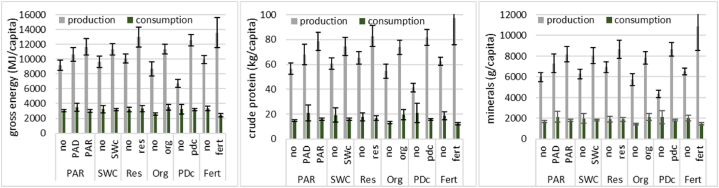
Per capita calorie, protein, and mineral nutrient production and own consumption from 15 crops in the Northern Highlands of Tanzania NB: PAR/D = participatory action research/demonstration; no = non-participants/non-users; SWC = soil and water conservation (y/n); res = residue incorporation (y/n); org = organic manure (y/n); pdc = pest and disease control; fert = fertilizer (y/n); minerals include calcium, magnesium, phosphorus, potassium, sodium, sulphur, and chlorine.

Total dietary energy, proteins, and minerals produced and consumed vary within the engagement level and SFM use (Fig. 2). Farmers who were fully engaged in action research (received fertilizer, modern improved seed, and training) yielded significantly more nutritional values than non-participants (27 % more calories, 39 % proteins, and 37 % minerals). However, those who were partially engaged in demonstration, though having higher production (16 % calories, 20 % proteins, 22 % minerals more), did not significantly differ from non-participants.

The SWC measures were implemented by 51 % of the farming households have a significant positive shift in dietary yields. Those that implemented SWC had 18 % calories, 22 % proteins, and 28 % minerals more than those that did not. Around 14 % of the farmers incorporated crop residues and their mean dietary outcomes are higher and significantly different from those that did not incorporate them. Their calories output was higher by 29 %, proteins by 27 %, and minerals by 25 %.

Farmers who applied manure (i.e., 68 % of the farmers) had significantly higher production of calories (31 % more, proteins (35 %), and minerals (36 %). The mean dietary yields under manuring were less variable (small standard error), indicating yield stabilisation effects. Those that applied manure consumed more of their own produced calories (34 %), proteins (49 %), and minerals (45 %) than those that did not apply. Sixty-five percent of farmers who controlled pests and diseases had higher per capita mean energy, protein, and mineral output than those that did not control.

Farmers who applied purchased fertilizer (i.e., 15 % of all farmers) had significantly higher production of calories (36 %), proteins (56 %), and minerals (67 %) than those that did not apply. This does not include those who applied free fertilizer because they are accounted for in participation in action research and demonstration. With less production, those that did not apply fertilizer significantly consume their production: 28 % calories, 34 % proteins, 27 % minerals more than those that applied. Farmers who adopted modern varieties had significantly higher outputs of calories (42 %), proteins (31 %), and minerals (27 %). These farmers constitute 30 % of all farmers and used modern improved varieties that were released after the initiation of action research in 2013.

### 3.2 Extended regression model (ERM) empirical results

The results from the ERM (i.e., estimation of equations (5)–(7)) show that the farmgate dietary outcomes for farmers who participated in the action research were significantly higher than non-participants (Table 2). The results for the 18-sub equations of determinants of participation & SFM (manure & fertilizer) are presented in the appendix Table 3. The Wald Chi-Squared Test that the explanatory variables, in addition to independently contributing to the model, the set is collectively significant. The first part of Table 2 for the ATTs shows that, compared to non-participants, per-capita calories, proteins, and minerals produced by farmers who were fully engaged in action research increased by 139, 216, and 143 % while for those that were partially engaged by 40, 54 and 42 %, respectively. The study further reveals significant differences in consumption of own-produced minerals. Farmers that fully participated in action research had increased mineral consumption by 208 % while those that were engaged in participatory action demonstration mineral consumption significantly increased by 70 %.

**Table 2:** Impact of participation in action research and associated technologies on per capita dietary outputs.

	production			consumption		
VARIABLES	energy	protein	minerals	energy	protein	minerals
**Treatment**						
PAD	1.40*	1.54***	1.42**	1.33	1.29	1.70*
0 = no,1 = yes	(0.26)	(0.22)	(0.21)	(0.41)	(0.55)	(0.54)
PAR	2.39**	3.16***	2.43***	2.04	1.82	3.08*
0 = no,1 = yes	(0.84)	(0.83)	(0.67)	(1.22)	(1.54)	(1.85)
**Endogenous**						
Inorganic fertilizer	1.61	1.68*	2.05***	0.82	1.15	1.68
0 = no,1 = yes	(0.56)	(0.49)	(0.48)	(0.26)	(0.99)	(0.67)
Organic manure	1.62***	1.73***	1.66***	2.13***	1.66***	1.94***
0 = no,1 = yes	(0.24)	(0.26)	(0.22)	(0.42)	(0.20)	(0.28)
**Other factors**						
Crop richness	1.17***	1.13***	1.11***	1.25***	1.17***	1.20***
number	(0.05)	(0.04)	(0.04)	(0.05)	(0.05)	(0.05)
Soil & water conservation	0.86**	0.87**	0.92	0.92	0.95	0.98
0 = no,1 = yes	(0.06)	(0.05)	(0.06)	(0.06)	(0.05)	(0.06)
Residue incorporation	0.92	0.92	0.94	0.97	0.95	0.97
0 = no,1 = yes	(0.11)	(0.10)	(0.10)	(0.10)	(0.08)	(0.09)
Pest & disease control	1.12	1.15*	1.17**	0.86*	0.87*	0.87*
0 = no,1 = yes	(0.09)	(0.09)	(0.09)	(0.07)	(0.06)	(0.07)
Labour availability	0.78***	0.78***	0.78***	0.84***	0.86***	0.85***
man-equivalent	(0.02)	(0.02)	(0.02)	(0.02)	(0.01)	(0.02)
Farm size	4.15***	3.81***	3.82***	2.01***	1.77***	1.74***
ha	(0.51)	(0.45)	(0.44)	(0.26)	(0.24)	(0.24)
Farm size squared	0.996**	0.997	0.997*	0.997**	0.998**	0.997**
	(0.002)	(0.002)	(0.002)	(0.001)	(0.001)	(0.001)
**Instrumental variables**						
Tropical livestock	1.00	0.999	0.999	1.00	1.001	1.001
index	(0.002)	(0.001)	(0.001)	(0.002)	(0.001)	(0.002)
Infor media access	1.03	1.02	0.97	1.17	1.17	1.09
0 = no,1 = yes	(0.15)	(0.13)	(0.12)	(0.14)	(0.20)	(0.15)
Constant	1781***	11.5***	1354***	696***	5.6***	396***
	(440)	(2.4)	(277)	(250)	(2.7)	(150)
**Correlations**						
Treatment, Outcome	−0.49 (0.14)***	−0.59 (0.09)***	−0.52 (0.11)***	−0.44 (0.26)*	−0.45 (0.46)	−0.65 (0.18)***
Endog_fert, Outcome)	−0.48 (0.18)***	−0.58 (0.12)***	−0.56 (0.11)***	−0.38 (0.27)	−0.49 (0.65)	−0.73 (0.19)***
Endog_org,Outcome)	−0.60 (0.11)***	−0.68 (0.08)***	−0.63 (0.09)***	−0.60 (0.17)***	−0.55 (0.28)**	−0.69 (0.15)***
Endog_fert, Treatment)	0.97 (0.03)***	0.96 (0.04)***	0.97 (0.02)***	0.96 (0.03)***	0.97 (0.03)***	0.97 (0.03)***
Endog_org,Treatment)	0.76 (0.17)***	0.85 (0.09)***	0.72 (0.18)***	0.69 (0.31)**	0.71 (0.43)*	0.77 (0.25)***
(Endog_org,Endog_fert)	0.77 (0.11)***	0.82 (0.06)***	0.75 (0.13)***	0.71 (0.21)***	0.73 (0.26)***	0.78 (0.15)***
Log *likelihood*	*−1634.16*	*−1594.91*	*−1586.51*	*−1631.49*	*−1497.83*	*−1544.20*
*Wald chi2(13)*	*520.62*	*504.72*	*521.79*	*220.38*	*260.85*	*176.59*
*Prob > chi2*	***	***	***	***	***	***
**Observations (n)**	553	553	553	549	549	549

Values in parenthesis are standard errors, *p < 0.1, **p < 0.05 ***p < 0.01. The coefficients are exponentiated and the percentage change is estimated as (β-1) *100.

**Table 3:** The extended regression model's probit sub-model results for determinants of participation and adoption.

	Production									Consumption								
	Eq (1).energy			Eq (2).protein			Eq (3).minerals			Eq (4).energy			Eq (5).protein			Eq (6).minerals		
VARIABLES	PAR	FERT	ORG	PAR	FERT	ORG	PAR	FERT	ORG	PAR	FERT	ORG	PAR	FERT	ORG	PAR	FERT	ORG
PAR		−1.99***	−1.62***		−1.92***	−1.88***		−2.00***	−1.51***		−1.94***	−1.39*		−1.99***	−1.47		−1.98***	−1.60**
		(0.16)	(0.46)		(0.18)	(0.25)		(0.14)	(0.49)		(0.16)	(0.84)		(0.17)	(1.14)		(0.17)	(0.68)
PA Demo		−0.93***	−0.91***		−0.91***	−1.04***		−0.93***	−0.85***		−0.90***	−0.75*		−0.92***	−0.80		−0.92***	−0.86**
		(0.10)	(0.25)		(0.11)	(0.14)		(0.09)	(0.27)		(0.10)	(0.45)		(0.11)	(0.59)		(0.10)	(0.36)
Crops grown	−0.08	−0.04	0.09	−0.08*	−0.04	0.07	−0.07	−0.04	0.10	−0.08*	−0.04	0.09	−0.08*	−0.03	0.09	−0.09*	−0.04	0.08
	(0.05)	(0.05)	(0.06)	(0.05)	(0.05)	(0.06)	(0.05)	(0.05)	(0.06)	(0.05)	(0.06)	(0.08)	(0.05)	(0.06)	(0.09)	(0.05)	(0.05)	(0.08)
Labour	2.3e-3	0.02	−0.01	2.2e+3	0.02	1.2e-3	2.6e-3	0.02	−0.01	3.5e-3	0.01	3.4e-3	−0.01	0.01	−0.01	−0.01	0.01	−0.01
	(0.03)	(0.03)	(0.03)	(0.03)	(0.03)	(0.03)	(0.03)	(0.03)	(0.03)	(0.03)	(0.03)	(0.03)	(0.03)	(0.03)	(0.03)	(0.03)	(0.03)	(0.03)
Cultivated land	0.16	0.21	0.39**	0.16	0.20	0.37**	0.16	0.21	0.43**	0.15	0.24	0.34*	0.15	0.21	0.34*	0.16	0.21	0.34*
	(0.12)	(0.14)	(0.17)	(0.12)	(0.14)	(0.15)	(0.12)	(0.14)	(0.17)	(0.12)	(0.15)	(0.17)	(0.12)	(0.15)	(0.18)	(0.12)	(0.13)	(0.17)
(ha)^2^		8.4e-4	−0.01**		7.7e-4	−0.01**		8.5e-4	−0.01**		1.0e-3	−0.01**		9.5e-4	−0.01*		1.1e-3	−0.01**
		(1.4e+3)	(2.4e+3)		(1.5e+3)	(2.1e+3)		(1.4e+3)	(2.6e+3)		(1.5e+3)	(2.8e+3)		(1.5e+3)	(3.3e+3)		(1.5e+3)	(2.7e+3)
tlu	3.7e+3	1.9e-3	0.07***	3.6e+3	1.4e-3	0.06***	3.4e+3	1.1e-3	0.07***	4.2e+3	4.0e-3	0.08**	4.1e+3	1.6e-3	0.08**	3.9e+3	6.7e-4	0.07**
	(2.7e+3)	(3.2e+3)	(0.02)	(2.5e+3)	(2.4e+3)	(0.02)	(2.6e+3)	(1.9e+3)	(0.03)	(2.7e+3)	(0.01)	(0.03)	(2.9e+3)	(5.0e+3)	(0.04)	(2.9e+3)	(1.8e+3)	(0.03)
Media access	0.27*	0.29*	0.19	0.25*	0.32*	0.21	0.27*	0.30*	0.20	0.27*	0.30*	0.16	0.28*	0.30*	0.16	0.28*	0.30*	0.17
	(0.15)	(0.17)	(0.17)	(0.15)	(0.17)	(0.16)	(0.15)	(0.17)	(0.18)	(0.15)	(0.17)	(0.18)	(0.16)	(0.17)	(0.19)	(0.15)	(0.16)	(0.17)
Education	0.04	0.16	0.23**	0.04	0.17*	0.21**	0.05	0.17*	0.25**	0.05	0.16	0.24**	0.07	0.19**	0.28**	0.04	0.17**	0.25**
	(0.10)	(0.10)	(0.11)	(0.09)	(0.10)	(0.10)	(0.09)	(0.10)	(0.10)	(0.10)	(0.10)	(0.11)	(0.10)	(0.10)	(0.13)	(0.09)	(0.09)	(0.11)
Age head	−0.03**	−0.01	4.5e-3	−0.03**	−0.01*	−0.01	−0.03**	−0.01**	4.7e-3	−0.02*	2.6e-3	1.9e+4	−0.02	2.5e-3	1.1e-3	−0.02	2.8e-3	6.6e-4
	(0.01)	(4.3e+3)	(3.9e+3)	(0.01)	(3.9e+3)	(3.5e+3)	(0.01)	(3.8e+3)	(4.1e+3)	(0.01)	(3.9e+3)	(4.2e+3)	(0.01)	(3.8e+3)	(4.7e+3)	(0.01)	(3.4e+3)	(3.9e+3)
(years)^2^	1.7e+4			1.6e+4			1.6e+4			1.9e+4			1.7e+4			1.7e+4		
	(1.2e+4)			(1.1e+4)			(1.2e+4)			(1.2e+4)			(1.3e+4)			(1.3e+4)		
Livestock_income_	−0.02	0.04*	0.09***	−0.03	0.04*	0.07**	−0.02	0.04*	0.09***	−0.03	0.03	0.08**	−0.02	0.03*	0.08	−0.02	0.04**	0.07*
	(0.02)	(0.02)	(0.03)	(0.02)	(0.02)	(0.03)	(0.02)	(0.02)	(0.03)	(0.02)	(0.02)	(0.04)	(0.02)	(0.02)	(0.06)	(0.02)	(0.02)	(0.04)
Nonfarm_income_	0.01	0.03	0.02	0.02	0.03*	0.02	0.01	0.03	0.02	3.5e+4	0.01	0.01	1.1e+4	0.02	0.01	3.9e+3	0.02	0.01
	(0.02)	(0.02)	(0.02)	(0.02)	(0.02)	(0.02)	(0.02)	(0.02)	(0.02)	(0.02)	(0.02)	(0.02)	(0.02)	(0.02)	(0.02)	(0.02)	(0.01)	(0.02)
Agent distance	1.1e-4***	6.4e-5*	1.1e-4***	1.2e-4***	6.4e-5*	1.1e-4***	1.2e-4***	7.3e-5**	1.2e-4***	8.7e-5*	4.0e-5	7.2e-5*	9.3e-5*	4.4e-5	8.3e-5**	6.9e-5*	2.1e-5	6.2e-5*
	3.1e+5	(3.5e+5)	(3.2e+5)	(2.9e+5)	(3.4e+5)	(2.8e+5)	(3.0e+5)	(3.2e+5)	(3.1e+5)	(4.6e+5)	(4.9e+5)	(3.9e+5)	(5.0e+5)	(5.7e+5)	(4.1e-4)	(3.9e+5)	(3.7e+5)	(3.6e+5)
Altitude	1.2e-3***		5.6e-5	1.2e-3***		3.3e-4	1.1e-3***		5.6e+5	1.3e-3***		1.5e-4	1.2e-3***		2.0e-4	1.2e-3***		2.2e-4
	(1.7e+4)		(3.4e+4)	(1.7e+4)		(2.4e+4)	(1.7e+4)		(3.4e+4)	(1.6e+4)		(4.6e+4)	(1.9e+4)		(6.3e+4)	(2.1e+4)		(4.4e+4)
Constant		0.39	0.53		0.34	1.24**		0.49	0.26		0.17	0.27		0.17	0.40		0.16	0.52
		(0.35)	(0.96)		(0.36)	(0.61)		(0.33)	(0.99)		(0.36)	(1.45)		(0.39)	(2.00)		(0.35)	(1.34)

Tlu = tropical livestock units; PAR = participatory action research; FERT = inorganic fertilizer; ORG = organic manure; the esimates are coeffiencts; Standard errors in parentheses; ***p < 0.01, **p < 0.05, *p < 0.1.

The results also show that the application of inorganic fertilizer increased the production of protein and minerals by 68 % and 105 % respectively. Organic manure application was associated with increased production and consumption of the three dietary food value groups by 62–113 %. Other production factors associated with enhanced production and consumption include crop diversity and farm size. Crop richness as an indicator of diversification increases calorie, protein and mineral production and consumption by 11–25 %. Farmers who took measures to control pests and diseases had significantly higher production by 12–17 % but the consumption was reduced by 13–14 %. The results also show that for a unit increase in farm size, calorie, protein, and mineral production increases by 3.8–4.2 % and consumption by 1.7–2.0 %. Soil and water conservation measures significantly reduced the production of energy and proteins by 14 and 13 %. Not conforming to the widely established positive labour-productivity relationship, the study found that labour availability reduced per-capital production and consumption of dietary food values by 22 and 15 % respectively.

The last part of Table 2 shows the correlation coefficients of the estimated equations. The results show that all the coefficients are significantly different from zero, implying there is endogenous treatment and sample selection, hence biased estimates would have been obtained if the study did not account for sample selection, endogenous treatment of participation in action research and the endogenous SFM adoption. In the case of the treatment and outcome equations, the results indicate that unobserved farmers' factors that reduce the participation in action research tend to also reduce dietary outcomes.

## 4 Discussion

Since 1980s, progress in the agricultural development using technologies and methods developed for other regions did not yield expected results in sub-Saharan Africa due to low technology adoption rates associated with suboptimal investments in extension services. Researchers stepped out and engaged farmers and extension agents in evaluating promising technologies suited to their areas. However, some studies found that in most supposedly participatory research programs involving vulnerable populations such as smallholder farmers, the socio-political power to decide on technologies rests with researchers and extension agents following scientific theory, principles, and design [60,61]. This study in addressing the question: ‘*how do farmers benefit from participation in action research?*‘, used production and consumption of dietary food values, and found that farmers who participated had higher calorie, protein and mineral production and intake.

There is growing evidence demonstrating the significance of participatory action research in promoting soil and production enhancing technologies such as improved germplasm and crop associations [2,60,62] which have been found to influence smallholder economic returns [[21], [63], [64]] and reduction in food insecurity [1,65]. This study provides further evidence that participation in action research contributes to outcomes indirectly through input and technology provision and but more importantly, directly through the development of human capital which collaborates with Sieber et al. [66]. Action research intends to engage farmers in questioning the existing knowledge and explore opportunities for the novelty to improve their farming practices [42]. As such, action research captivates action through innovativeness, fostering (re)search and (un)learning cycle and perpetuating the quest for improvement. From the results, it is observed that impact of full engagement in action research through the implementation of multi-technology evaluation trials with researcher support is higher than the partial engagement in demonstration. Farmers’ engagement in choice and testing of interventions in research for development has been found to bolster the use of optimal solutions for smallholder farm conditions. This is attributed to increased knowledge, record keeping for informed farm decisions and their capacity to implement improved technologies [67]. Downstream, participation in action research reinforces farmer-to-farmer technology and knowledge transfer and market linkage [60].

Inorganic fertilizer and manure were the two main endogenous soil fertility management technologies promoted under Africa RISING [68]. The endogenous effect of organic manure shows a large and wide-ranging impact on dietary outcomes. Large effects of organic manure on food security were also found in a study by Asfaw et al. [69] in Niger. In these low resource agro-pastoral systems, the wide usage of organic manure indicates their reliance on the locally available nutrient source and attempts to recycle resources in the farming systems [48]. The results also signify that subsistence-oriented farmers tend to rely on organic farming strategies [70].

Despite targeting maize as the main cereal crop, other crops benefit directly from fertilisation in the intercrop or from the residual nutrient supplies. There is overwhelming evidence that fertilisation increases crop yields in the tropics by 50–60 % thereby reducing food insecurity [69,71,72]. However, in Tanzania, the use of inorganic fertilizers was affected by the boom and burst of the 1980s [48]. Our study did not detect evidence of fertilizer's impact on dietary consumption but affects protein and mineral outputs and is in line with the evidence that fertilisation in smallholder farming systems contributes to food and nutrition production.

In a Ghanaian study, Nata et al. [72] found that farmers who reported pest infestation and pesticide use had a lower probability of being food secure. The study use farmers' effort to control pests and diseases and establish that although the practice enhances the production of proteins and minerals, it lowers the dietary consumption from own production. The plausible reason could be that farmers mostly sell to recover pest and disease control expenditures.

The negative effect of SWC's role in decreasing dietary food value production contrasts with anecdotal experiences by Tanzanian farmers who attributed a decrease in soil erosion, increase in crop yields and safeguarding food sufficiency to SWC [73,74]. The result further contradicts with the evidence generated by Sileshi et al. [75] in Ethiopia who found that the restoration measures support positive net crop produce value thereby reducing the probability of farmers being food insecure. Our results show that investments in SWC which mostly include physical structures and long-term vegetative measures, with primary purpose of reclaiming degraded lands, takes time to yield results and has initial cost to the farmers engaged that would result in food insecurity.

The results also reveal that the impacts of participation in action research and adoption of soil fertility management practices on dietary outcomes are co-determined by farm and farmer specific attributes. Land size as a major household resource has a strong positive influence on per capita production and consumption while labour availability imposes a constraint. Although inverse farm-size productivity relationships have been observed since the early 2000s [76], larger land sizes support per-capita production [77] and our findings show that those with limited land have significantly lower production and produce consumption levels. Although labour is generally regarded as a constraint, in smallholder farming systems, farmers manage considerably small plots and tend to have unlimited labour supplies. Lewis [78] purports that labour allocation behaviour for subsistence farmers cannot be fully explained by capitalistic theories that assume competitiveness as there is some level of unawareness on the margins from different labour investment portfolios. In rural areas with unskilled labour, poor soils and variable production, there is a high level of factor substitutability and disutility among members after extended engagement in farming renders them to allocate less labour commensurate to marginal productivity [79].

## 5 Conclusion and policy implications

This study assesses the impacts of participation in PAR and adoption of agronomic technologies on dietary outcomes in Tanzania using data from over 600 households. The decision to participate in action research and adopt SFM technologies may however be endogenous as farmers usually self-select into action research program/ SFM adoption based on both observable and unobservable characteristics. Without controlling for this, the effects of participation and SFM adoption on dietary outcomes would be biased. To account for the potential endogeneity of participation and the adoption of SFM technologies, the ERM is used.

Results indicate that engagement in PAR increases farmers' agronomic knowledge and practices with a significant impact on farm-gate dietary outcomes. Farmers participating in PAR produce and consume more calories, proteins, and minerals than their non-participant counterparts, after accounting for covariates and endogeneity. Among the endogenous soil fertility measures, locally sourced organic manure positively impacts both production and consumption while purchased inorganic fertilizers influence only production. Land and labour have inverse impacts: while large landholdings support per capita dietary production and consumption, farming households with more labour do not exploit for farming. These findings indicate that participatory action research has the potential to improve household food and nutritional security and further highlight the need for a strategic focus on promoting crop diversification and organic manure to enable sustained productivity of resource-constrained smallholdings with minimal labour inputs.

Although the study accounted for self-selection, reverse causality and omitted variable bias using quasi-experimental instrumental variable approach and potential endogeneity and simultaneity using extended regression model, it does not fully address the challenges of ascertaining the validity of causal inference made with observational program impact evaluation. Hence, these findings are indicative of potential impact and requires further validation with robust approaches.

## 6 Limitations of the study

Our study has some limitations within which the findings need to be interpreted carefully. First, as in most empirical studies, research was limited by approaches due to uncorrelated factors, feedback loops and system dynamics, the causal chain is hard to come by. Secondly, our study was cross-sectional in nature and assess respondents’ perception of the PAR engagement at a specific time and location. Third, although the study was conducted in an archetypal smallholder setting, the results may not be completely generalisable because the sample was restricted to Babati District in Tanzania. Last but not least, the study did not analyse the impact of random assignment of the participation, hence the results are pointers and need to be validated with robust study designs.

## Ethical clearance

The research tools and procedures for data collection, analysis, management, and sharing were reviewed by the Institutional Review Board (IRB) of the Alliance of Bioversity International and CIAT which waived a full review with reference: Africa RISING (2021-IRB22).

## Data availability

The data associated with this study has not been deposited into a publicly available repository. It will be made available from the author, JK, upon reasonable request.

## CRediT authorship contribution statement

**Powell Mponela:** Writing – review & editing, Writing – original draft, Methodology, Formal analysis, Data curation, Conceptualization. **Julius Manda:** Writing – review & editing, Methodology, Conceptualization. **Michael Kinyua:** Writing – review & editing, Project administration, Investigation, Data curation. **Job Kihara:** Writing – review & editing, Supervision, Project administration, Investigation, Funding acquisition, Conceptualization.

## Declaration of competing interest

All authors declare that they have no conflicts of interest.

## References

[1] Kangmennaang J., Kerr R.B., Lupafya E., Dakishoni L., Katundu M., Luginaah I. (2017). Impact of a participatory agroecological development project on household wealth and food security in Malawi. Food Secur..

[2] Snapp S., Dedecker J., Davis A.S. (2019). Farmer participatory research advances sustainable agriculture: lessons from Michigan and Malawi. Agron. J..

[3] Van Vugt D., Franke A.C., Giller K.E. (2017). PARTICIPATORY RESEARCH to CLOSE the SOYBEAN YIELD GAP on SMALLHOLDER FARMS in Malawi. Exp. Agric..

[4] Sseguya H., Robinson D.S., Mwango H.R., Flock J.A., Id J.M., Abed R., Mruma S.O. (2021).

[5] Mponela P., Manda J., Kinyua M., Kihara J. (2022). Participatory action research, social networks, and gender influence soil fertility management in Tanzania. Syst. Pract. Action Res..

[6] Manda J., Azzarri C., Feleke S., Kotu B., Claessens L., Bekunda M. (2021). Welfare impacts of smallholder farmers' participation in multiple output markets: empirical evidence from Tanzania. PLoS One.

[7] Rusike J., Snapp S., Twomlow S.J. (1998). Mother-baby trial approach for developing soil water and fertility management technologies. Particip. Res. Dev. Sustain. Agric. Nat. Resour. Manag. A Sourceb..

[8] Abebe G. (2005). Participatory selection of drought tolerant maize varieties using mother and baby methodology: a case study in the semi arid zones of the Central Rift Valley of Ethiopia. Afr. Crop Sci. Conf. Proc..

[9] Buah S., Kombiok J., Kanton R., Denwar N., Haruna A., Wiredu A., Abdulai M. (2013). Participatory evaluation of drought tolerant maize varieties in the Guinea Savanna of Ghana using mother and baby trial design. J. Sci. Technol..

[10] Reddy K.S., Mohanty M., Rao D.L.N., Rao A.S., Pandey M., Singh M., Dixit S.K., Dalal R.C., Blamey F.P.C., Menzies N.W. (2015). Farmer involvement in the development and adoption of improved nutrient management technologies using the mother–baby trial approach in vertisols. Proc. Natl. Acad. Sci. India B Biol. Sci..

[11] Mapfumo P., Adjei-Nsiah S., Mtambanengwe F., Chikowo R., Giller K.E. (2013). Participatory action research (PAR) as an entry point for supporting climate change adaptation by smallholder farmers in Africa. Environ. Dev..

[12] Snapp S., BELLON M.R., REEVES J. (2002). Quant. Anal. Data from Particip. Methods Plant Breed., Experimental Agriculture.

[13] Dixon J., Gulliver A., Gibbon D. (2001). http://www.fao.org/3/a-ac349e.pdf.

[14] Anderson J., Marita C., Musiime D. (2016).

[15] URT (2014). https://www.kilimo.go.tz/index.php/en/resources/view/tanzania-agriculture-climate-resilience-plan-20142019.

[16] Arndt C., Farmer W., Strzepek K., Thurlow J. (2012). Climate change, agriculture and food security in Tanzania. Rev. Dev. Econ..

[17] Pauw K., Thurlow J. (2011). Agricultural growth, poverty, and nutrition in Tanzania. Food Pol..

[18] Cochrane N., D'Souza A. (2015). https://www.ers.usda.gov/webdocs/publications/43932/51864_eib135.pdf?v=9954.3.

[19] Vanlauwe B., Bationo A., Chianu J., Giller K.E., Merckx R., Mokwunye U., Ohiokpehai O., Pypers P., Tabo R., Shepherd K.D., Smaling E.M.A., Woomer P.L., Sanginga N. (2010). Integrated soil fertility management; Operational definition and consequences for implementation and dissemination. Outlook Agric..

[20] Barrett C.B., Bevis L.E.M. (2015). The self-reinforcing feedback between low soil fertility and chronic poverty. Nat. Geosci..

[21] Hörner D., Wollni M. (2021). Integrated soil fertility management and household welfare in Ethiopia. Food Pol..

[22] Azzarri C., Haile B., Letta M. (2022). Plant different, eat different? Insights from participatory agricultural research. PLoS One.

[23] V Santoso M., Bezner Kerr R.N., Kassim N., Martin H., Mtinda E., Njau P., Mtei K., Hoddinott J., Young S.L. (2021). A nutrition-sensitive agroecology intervention in rural Tanzania increases children's dietary diversity and household food security but does not change child anthropometry: results from a cluster-randomized trial. J. Nutr..

[24] Kerr R.B., Berti P.R., Shumba L. (2011). Effects of a participatory agriculture and nutrition education project on child growth in northern Malawi. Publ. Health Nutr..

[25] Ogutu S.O., Gödecke T., Qaim M. (2019). Agricultural commercialisation and nutrition in smallholder farm households. J. Agric. Econ..

[26] Tandon R., de Koning K., Martin M. (1996). Particip. Res. Heal. Issues Exp..

[27] Fao Tanzania (2018). https://www.fao.org/3/I8356EN/i8356en.pdf.

[28] Peter K.H., Nnko H.J., Mubako S. (2020). Impacts of anthropogenic and climate variation on spatiotemporal pattern of water resources: a case study of Lake Babati, Tanzania. Sustain. Water Resour. Manag..

[29] Kihara J., Tamene L., Massawe P., Bekunda M. (2015). Agronomic survey to assess crop yield, controlling factors and management implications: a case-study of Babati in northern Tanzania. Nutrient Cycl. Agroecosyst..

[30] Mugi-Ngenga E., Zingore S., Bastiaans L., Anten N.P.R., Giller K.E. (2021). Farm-scale assessment of maize – pigeonpea productivity in Northern Tanzania. Nutrient Cycl. Agroecosyst..

[31] Ekblom M. (2016). https://www.diva-portal.org/smash/get/diva2:935700/FULLTEXT01.pdf.

[32] Venance S.K., Mshenga P., Birachi E. (2016). Factors influencing on-farm common bean profitability : the case of smallholder bean farmers in Babati District , Tanzania. J. Econ. Sustain. Dev..

[33] Njuguma C. (2014). https://africa-rising.net/dietary-and-income-diversification/.

[34] Rajendran S., Afari-Sefa V., Shee A., Bocher T., Bekunda M., Dominick I., Lukumay P.J. (2017). Does crop diversity contribute to dietary diversity? Evidence from integration of vegetables into maize-based farming systems. Agric. Food Secur..

[35] Khonje M.G., Manda J., Mkandawire P., Tufa A.H., Alene A.D. (2018). Adoption and welfare impacts of multiple agricultural technologies: evidence from eastern Zambia. Agric. Econ..

[36] Ogundari K., Bolarinwa O.D. (2019). Does adoption of agricultural innovations impact farm production and household welfare in sub-saharan Africa? A meta-analysis. Agric. Resour. Econ. Rev..

[37] Manda J., Azzarri C., Feleke S., Kotu B., Claessens L., Bekunda M. (2021). Welfare impacts of smallholder farmers' participation in multiple output markets: empirical evidence from Tanzania. PLoS One.

[38] Wordofa M.G., Hassen J.Y., Endris G.S., Aweke C.S., Moges D.K., Rorisa D.T. (2021). Adoption of improved agricultural technology and its impact on household income: a propensity score matching estimation in eastern Ethiopia. Agric. Food Secur..

[39] Rutterford C., Copas A., Eldridge S. (2015).

[40] Wunsch G., Russo F., Mouchart M. (2010). Do we necessarily need longitudinal data to infer causal relations?. Bull. Sociol. Methodol. Méthodologie Sociol..

[41] Cox D.R. (1992). Causality: some statistical aspects. J. R. Stat. Soc. Ser. A (Statistics Soc..

[42] Baum F., Macdougall C., Smith D. (2006).

[43] Wooldridge J.M. (2010).

[44] Nahayo A., Omondi M.O., Zhang X., Li L., Pan G., Joseph S. (2017). Factors influencing farmers' participation in crop intensification program in Rwanda. J. Integr. Agric..

[45] Pattanayak S.K., Evan Mercer D., Sills E. (2003). Taking stock of agroforestry adoption studies. Agrofor. Syst..

[46] Beyene A.D., Kassie M. (2015). Speed of adoption of improved maize varieties in Tanzania: an application of duration analysis. Technol. Forecast. Soc. Change.

[47] FAO, FAO/ INFOODS (2017).

[48] Löfstrand F. (2005). https://stud.epsilon.slu.se/11867/1/lofstrand_f_171013.pdf.

[49] Pizer S.D. (2016). Falsification testing of instrumental variables methods for comparative effectiveness research. Health Serv. Res..

[50] McArthur J.W., McCord G.C. (2017). Fertilizing growth: agricultural inputs and their effects in economic development. J. Dev. Econ..

[51] Angrist J.D., Krueger A.B. (2001). Instrumental variables and the search for identification: from supply and demand to natural experiments. J. Econ. Perspect..

[52] Pan Y., Smith S.C., Sulaiman M. (2018). Agricultural extension and technology adoption for food security: evidence from Uganda. Am. J. Agric. Econ..

[53] Cawley A., O'Donoghue C., Heanue K., Hilliard R., Sheehan M. (2018). The impact of extension services on farm‐level income: an instrumental variable approach to combat endogeneity concerns. Appl. Econ. Perspect. Pol..

[54] Jaleta M., Kassie M., Tesfaye K., Teklewold T., Jena P.R., Marenya P., Erenstein O. (2016). Resource saving and productivity enhancing impacts of crop management innovation packages in Ethiopia. Agric. Econ..

[55] Zeng D., Alwang J., Norton G.W., Shiferaw B., Jaleta M., Yirga C. (2017). Agricultural technology adoption and child nutrition enhancement: improved maize varieties in rural Ethiopia. Agric. Econ..

[56] Abdoulaye T., Wossen T., Awotide B. (2018). Impacts of improved maize varieties in Nigeria: ex-post assessment of productivity and welfare outcomes. Food Secur..

[57] Kubitza C., Krishna V.V. (2020). Instrumental variables and the claim of causality: evidence from impact studies in maize systems. Global Food Secur..

[58] Statacorp (2021). https://www.stata.com/manuals/erm.pdf.

[59] Cox D.R., Wermuth N. (2001). Some statistical aspects of causality. Eur. Socio Rev..

[60] Olarinde L., Binam J., Fatunbi A.O., Diagne A., Adekunle A., Ayanwale A. (2017). Participatory research demonstration and its impact on the adoption of improved agricultural technologies in the savannas of West Africa. Afr. Crop Sci. J..

[61] Cook B.R., Satizábal P., Curnow J. (2021). Humanising agricultural extension: a review. World Dev..

[62] Kpaka H.M., Wossen T., Stein D., Mtunda K., Laizer L., Feleke S., Manyong V. (2021). Rural schools as effective hubs for agricultural technology dissemination: experimental evidence from Tanzania and Uganda. Eur. Rev. Agric. Econ..

[63] Khonje M.G., Manda J., Alene A.D., Kassie M. (2015). Analysis of adoption and impacts of improved maize varieties in eastern Zambia. World Dev..

[64] Kim J., Mason N.M., Snapp S., Wu F. (2019). Does sustainable intensification of maize production enhance child nutrition? Evidence from rural Tanzania. Agric. Econ..

[65] Graef F., Uckert G., Schindler J. (2017). Expert-based ex-ante assessments of potential social, ecological, and economic impacts of upgrading strategies for improving food security in rural Tanzania using the ScalA-FS approach. Food Secur..

[66] Sieber S., Graef F., Mutabazi K.D., Tumbo S.D., Faße A., Paloma S.G.Y., Rybak C., Lana M., Ndah T.H., Uckert G., Schuler J., Grote U. (2018).

[67] Francis J., Sibanda S. (2001). Participatory action research experiences in smallholder dairy farming in Zimbabwe. Livest. Res. Rural Dev..

[68] Kihara J., Kizito F., Jumbo M., Kinyua M., Bekunda M. (2020). Unlocking maize crop productivity through improved management practices in northern Tanzania. Afr. J. Food Nutr. Sci..

[69] Asfaw S., Di Battista F., Lipper L. (2014).

[70] Kipsat M.J., Osewe D.O., Bwari M.P. (2021). Assessment of factors that limit optimal use of organic fertilizers in subsistence food production in vihiga county, Kenya. Int. J. Environ. Clim. Chang..

[71] Stewart W.M., Roberts T.L. (2012). Food security and the role of fertilizer in supporting it. Procedia Eng..

[72] Nata J.T., Mjelde J.W., Boadu F.O. (2014). Household adoption of soil-improving practices and food insecurity in Ghana. Agric. Food Secur..

[73] Shrestha R.P., Ligonja P.J. (2015). Social perception of soil conservation benefits in Kondoa eroded area of Tanzania. Int. Soil Water Conserv. Res..

[74] Habtemariam L.T., Mgeni C.P., Mutabazi K.D., Sieber S. (2019). The farm income and food security implications of adopting fertilizer micro-dosing and tied-ridge technologies under semi-arid environments in central Tanzania. J. Arid Environ..

[75] Sileshi M., Kadigi R., Mutabazi K., Sieber S. (2019). Impact of soil and water conservation practices on household vulnerability to food insecurity in eastern Ethiopia: endogenous switching regression and propensity score matching approach. Food Secur..

[76] Matchaya G.C. (2007). Does size of operated area matter? Evidence from Malawi's agricultural production. Int. J. Agric. Rural Dev..

[77] Dorward A. (1999). Farm size and productivity in Malawian smallholder agriculture. J. Dev. Stud..

[78] Lewis W.A. (1954). Economic development with unlimited supplies of labour. Manch. Sch..

[79] Gavish B., Kalay A. (1983). On the asset substitution problem. J. Financ. Quant. Anal..

